# Mixed Germ Cell Tumour in a Case of Pure Gonadal Dysgenesis (Swyer Syndrome) - A Case Report

**DOI:** 10.7759/cureus.459

**Published:** 2016-01-13

**Authors:** Naveen P Kumar, Venugopal M, Anitha Mathews, Francis V James

**Affiliations:** 1 Division of Radiation Oncology, Regional Cancer Centre Trivandrum; 2 Division of Imageology, Regional Cancer Centre Trivandrum; 3 Division of Pathology, Regional Cancer Centre Trivandrum

**Keywords:** swyer syndrome, germ cell tumour, mature teratoma

## Abstract

Swyer syndrome or pure gonadal dysgenesis 46, XY is a medical condition associated with 46 XY karyotype and primary amenorrhea in a phenotypic female. In this syndrome, there is an abnormality in testicular differentiation. Patients with disorders in sexual differentiation have an increased risk for development of genital malignancies. A 14-year-old female admitted with abdominal pain was diagnosed to have Swyer syndrome and a pelvic tumor after clinical and laboratory investigations. She underwent surgery, and the histology report revealed a mixed germ cell tumor in a dysgenetic gonad. She recurred three months later and was successfully treated with chemotherapy and a second surgery to remove the differentiated teratoma. The early diagnosis of patients with Swyer syndrome is important because of the increased risk for the development of malignancy. Early surgical treatment is required. Recurrent and metastatic disease respond well to chemotherapy.

## Introduction

Gonadal dysgenesis is defined as an incomplete or defective formation of the gonads, resulting from a disturbed process of migration of the germ cells and/or their correct organization in the fetal gonadal ridge. It is caused by structural or numerical anomalies of the sex chromosomes or mutations in one of the genes involved in the formation of the urogenital ridge and sex determination of the bipotential gonad [1]. Neoplastic transformation of germ cells in dysgenetic gonads (the formation of gonadoblastoma and/or an invasive germ cell tumor) occurs, according to literature, in 20–30% of cases and is associated with the presence of (part of) the Y chromosome in the patients’ karyotype [2-3]. It's usually diagnosed at a young age. Neoplasms have been discovered as early as six months of age [4].

Here, we report a case of mixed germ cell tumour diagnosed in a dysgenetic gonad in a 14-year-old girl with Swyer syndrome.

## Case presentation

**Figure 1:**
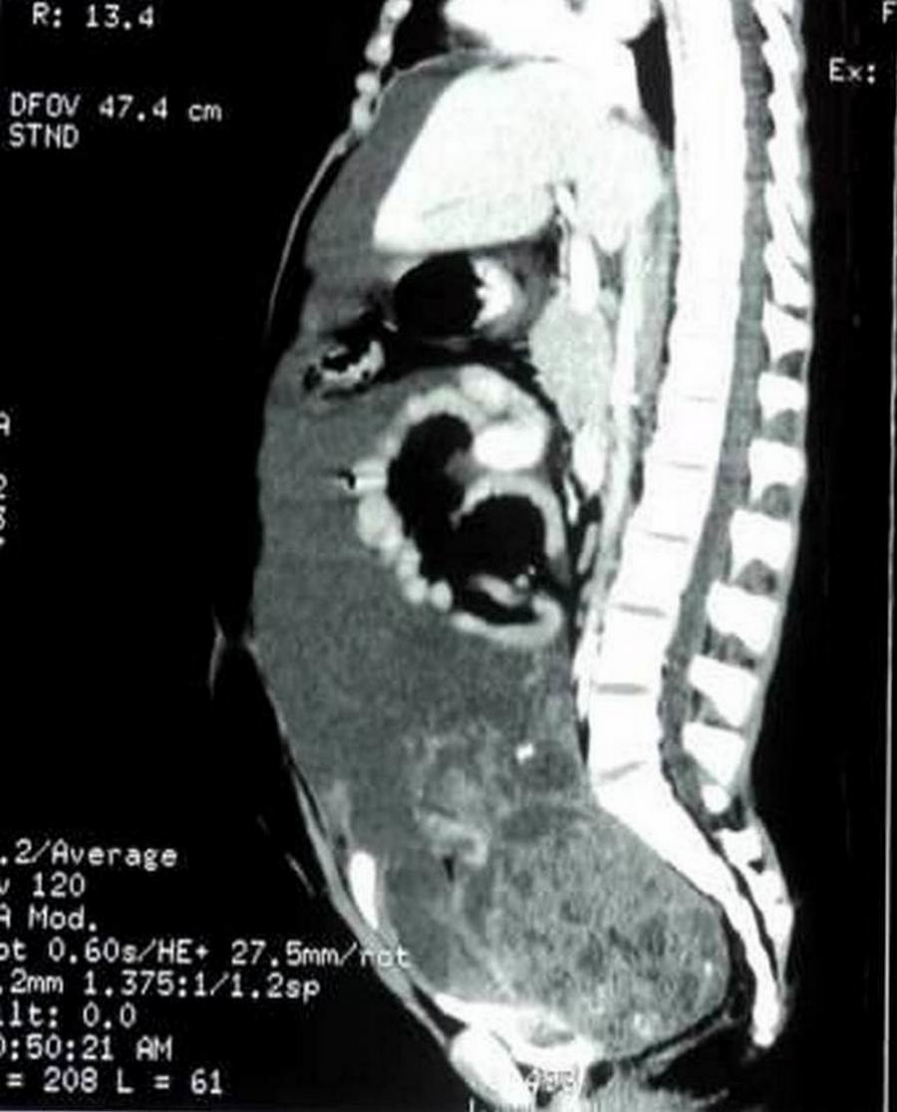
Initial preoperative imaging showing pelvi abdominal mass

**Figure 2:**
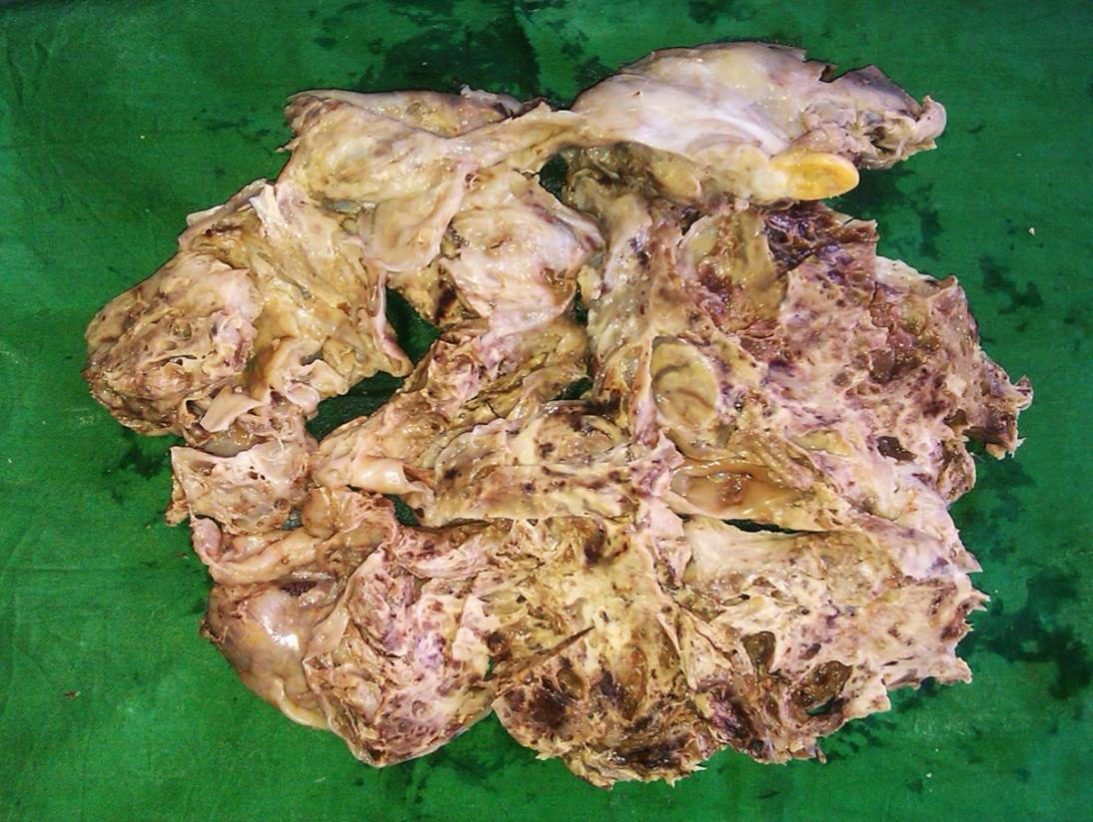
Gross specimen after total abdominal hysterectomy and salpingo oophorectomy

**Figure 3:**
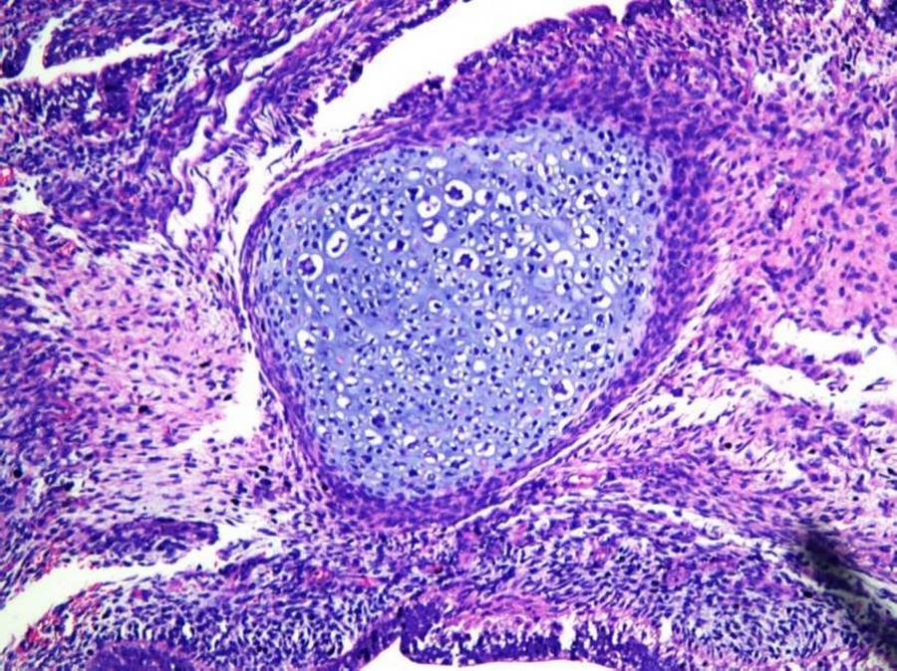
Teratomatous elements in the tumour

**Figure 4:**
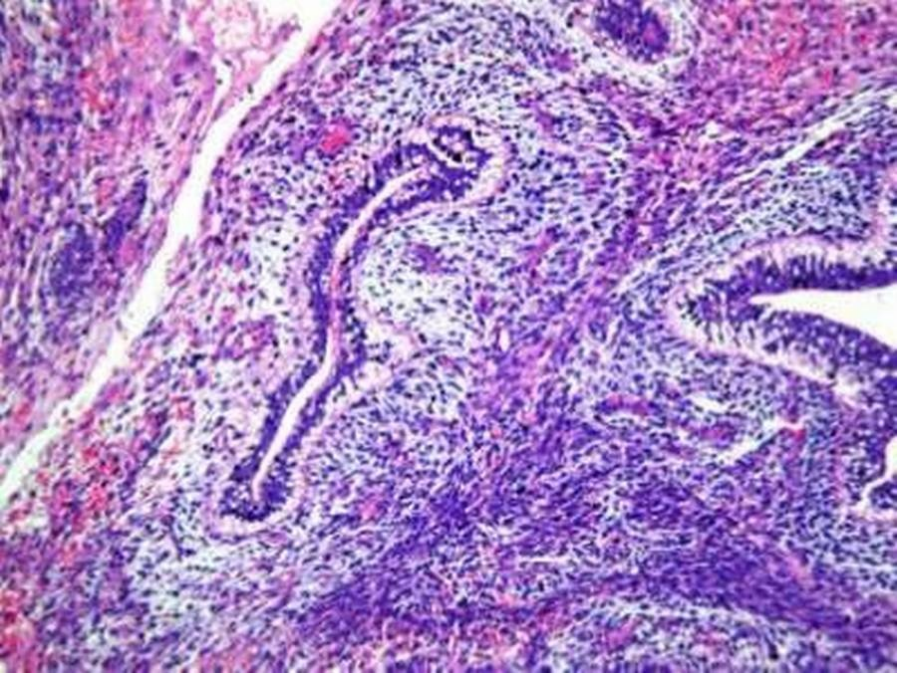
Yolk sac elements

**Figure 5:**
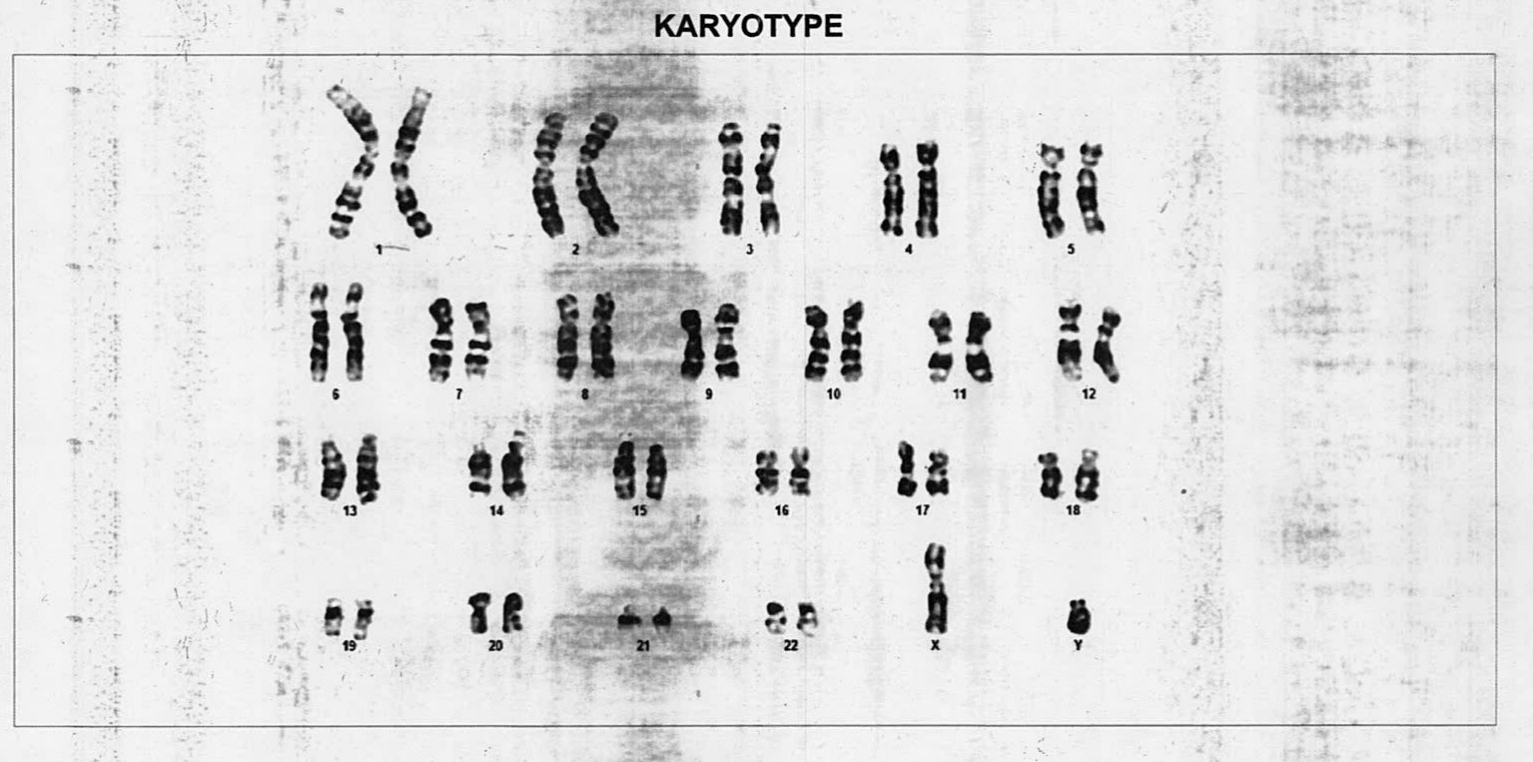
Karyotype showing 46, XY pattern

A 14-year-old phenotypic female, the eldest of three children born out of a non-consanguineous marriage and an uneventful, full-term normal delivery, presented to a local hospital with a one-month history of diffuse abdominal pain, progressive abdominal distention, and vomiting. Her past surgical and medical history was unremarkable. She was premenarchal and had no history of developmental delay. There was no family history of any malignancies or developmental anomalies. On physical examination, she was 155 cm tall and weighed 50 kg. She did not have any secondary sexual characters, and her external genitalia appeared to be that of a normal female phenotype. Her abdominal examination revealed a large pelvic-abdominal mass occupying almost the whole of the abdomen with gross ascites. Systemic examination was within normal limits. Imaging showed a large lobulated heterogeneous mass in the pelvis extending to the abdomen with numerous cystic areas (Figure 1). Baseline serum tumor markers were not available. With a provisional diagnosis of an ovarian tumor and informed consent, she underwent an exploratory laparotomy. Perioperatively it was seen that she had rudimentary Mullerian structures and a small fibrous tissue in place of the left ovary. The right ovary was the seat of a 22 x 20 x 13 cm tumor. A total abdominal hysterectomy with bilateral salpingo-oophorectomy and omentectomy were done. The final pathology report revealed a mixed germ cell tumor consisting of teratoma, dysgerminoma, and focal yolk sac components with a rudimentary uterus, tubes, and streak gonad on the left (Figures 2-4). Ascitic fluid and omentum were free of malignancy. Karyotyping showed 46, XY genotype (Figure 5).

**Figure 6:**
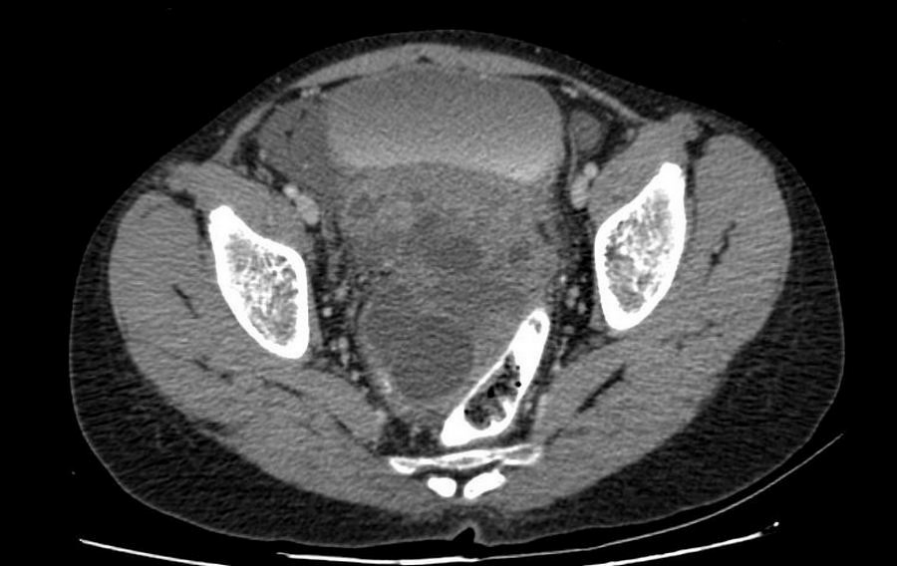
Residual lesion after four cycles of chemotherapy

She was referred to us but reported only three months later with progressive abdominal symptoms and raised levels of serum (AFP), beta-HCG, and LDH. A CT scan of the abdomen showed a huge pelvic recurrence. She received four cycles of combination chemotherapy with bleomycin, etoposide, and cisplatinum. At the end of four cycles, serum markers normalised, but she still had a large residual pelvic mass (Figure 6). She underwent a gross total removal of the residual tumor, which was teratomatous histologically. A postoperative CT scan of the abdomen two months after the surgery did not show any evidence of disease, and markers remained normal. She is currently being followed up once in three months with imaging and tumor marker assays and is doing well one and a half years after her last surgery.

## Discussion

The first reported case of Swyer syndrome was in 1955 by Dr G I Swyer who described two women who had a 46, XY karyotype, tall stature, primary amenorrhea, female external genitalia, normal vagina (albeit hypoestrogenized), and cervix [5]. Individuals with Swyer syndrome are phenotypically female with unambiguously female genital appearance from birth and hypoplastic to normal Müllerian structures. The patients first become apparent usually in adolescence with delayed puberty and primary amenorrhea due to the fact that the gonads have no hormonal or reproductive potential. As expected, they show elevated gonadotropins, normal female levels of androgens, and low levels of estrogens. Both gonads display fibrous tissue that vaguely resembles ovarian stroma but without follicles. The etiology of 46, XY pure gonadal dysgenesis is thought to be a short-arm Y chromosome deletion involving SRY (putative testicular-determining factor gene), a mutation in other genes that leads to inhibition of SRY function, or a mutation of SRY function [6]. 

During embryogenesis, without any external influences for or against, the human reproductive system is intrinsically conditioned to give rise to a female reproductive organization. As a result, if a gonad cannot express its sexual identity via its hormones (as in gonadal dysgenesis), then the affected person, no matter whether their chromosomes are XY or XX, will develop external female genitalia. Internal female genitalia, primarily the uterus, may or may not be present depending on the etiology of the disorder.

The incidence of neoplasia in patients with gonadal dysgenesis merits mention. Slowikowska et al. [7] reported that neoplasia can occur in 16.7% to 23.1% of patients with gonadal dysgenesis. In a clinical review of 194 cases of neoplasia in dysgenetic gonads, 103 (53.1%) were gonadoblastomas, 38 (19.6%) dysgerminomas, 34 (17.5%), gonadoblastoma with areas of dysgerminoma, and 19 (9.8%) were of other histologic types [4]. Therefore, in premenarchal patients with a pelvic mass, the karyotype should be determined.

The treatment of such a patient is primarily surgical, including resection of the primary lesion and proper surgical staging. Chemotherapy is administered to patients with recurrent and metastatic disease [8]. The common regimens used include Cisplatin, Ifosfamide, and Etoposide/Vinblastin/Paclitaxel (VIP/VeIP/TIP). Gemcitabine, Irinotecan, and Oxaliplatin based regimens are also used. Those who fail may be offered high-dose chemotherapy with autologous stem-cell rescue. Surgical salvage remains important in resectable, platinum refractory cases and in those with late relapses beyond two years

## Conclusions

Swyer syndrome or pure gonadal dysgenesis 46, XY is associated with a significant risk of developing germ cell tumors, and prophylactic gonadectomy is recommended. In any patient with a premenarchal pelvic mass, karyotyping should be performed. With prompt treatment, either surgery alone or surgery with chemotherapy, a good response and favorable survival outcomes can be obtained even in a metastatic or recurrent setting.

## References

[1] Cools M, Stoop H, Kersemaekers AM, Drop SL, Wolffenbuttel KP, Bourguignon JP, Slowikowska-Hilczer J, Kula K, Faradz SM, Oosterhuis JW, Looijenga LH ( 2006). Gonadoblastoma arising in undifferentiated gonadal tissue within dysgenetic gonads. J Clin Endocrinol Metab.

[2] Manuel M, Katayama PK, Jones HW Jr (1976). The age of occurrence of gonadal tumors in intersex patients with a Y chromosome. Am J Obstet Gynecol.

[3] Verp MS, Simpson JL (1987). Abnormal sexual differentiation and neoplasia. Cancer Genet Cytogenet.

[4] Troche V, Hernandez E (1986). Neoplasia arising in dysgenetic gonads. Obstet Gynecol Surv.

[5] Swyer GIM (1955). Male pseudohermaphroditism: a hitherto undescribed form. Br Med J.

[6] Behzadian MA, Tho SP, McDonough PG (1991). The presence of the testicular determining sequence, SRY, in 46, XY females with gonadal dysgenesis (Swyer syndrome). Am J Obstet Gynecol.

[7] Słowikowska-Hilczer J, Romer TE, Kula K (2003). Neoplastic potential of germ cells in relation to disturbances of organogenesis and changes in karyotype. J Androl.

[8] Behtash N, Karimi Zarchi M (2007). Dysgerminoma in three patients with Swyer syndrome. World J Surg Oncol.

